# PTPN2 regulates bacterial clearance in a mouse model of enteropathogenic and enterohemorrhagic *E*. *coli* infection

**DOI:** 10.1172/jci.insight.156909

**Published:** 2023-02-22

**Authors:** Marianne R. Spalinger, Vinicius Canale, Anica Becerra, Ali Shawki, Meli’sa Crawford, Alina N. Santos, Pritha Chatterjee, Jiang Li, Meera G. Nair, Declan F. McCole

**Affiliations:** 1 Division of Biomedical Sciences, School of Medicine, University of California, Riverside, Riverside, California, USA.; 2 Department for Gastroenterology and Hepatology, University Hospital Zurich and University of Zurich, Zurich, Switzerland.

**Keywords:** Gastroenterology, Inflammation, Bacterial infections, Cellular immune response, Macrophages

## Abstract

Macrophages intimately interact with intestinal epithelial cells, but the consequences of defective macrophage–epithelial cell interactions for protection against enteric pathogens are poorly understood. Here, we show that in mice with a deletion in protein tyrosine phosphatase nonreceptor type 2 (PTPN2) in macrophages, infection with *Citrobacter rodentium*, a model of enteropathogenic and enterohemorrhagic *E*. *coli* infection in humans, promoted a strong type 1/IL-22–driven immune response, culminating in accelerated disease but also faster clearance of the pathogen. In contrast, deletion of PTPN2 specifically in epithelial cells rendered the epithelium unable to upregulate antimicrobial peptides and consequently resulted in a failure to eliminate the infection. The ability of PTPN2-deficient macrophages to induce faster recovery from *C*. *rodentium* was dependent on macrophage-intrinsic IL-22 production, which was highly increased in macrophages deficient in PTPN2. Our findings demonstrate the importance of macrophage-mediated factors, and especially macrophage-derived IL-22, for the induction of protective immune responses in the intestinal epithelium, and show that normal PTPN2 expression in the epithelium is crucial to allow for protection against enterohemorrhagic *E*. *coli* and other intestinal pathogens.

## Graphical abstract

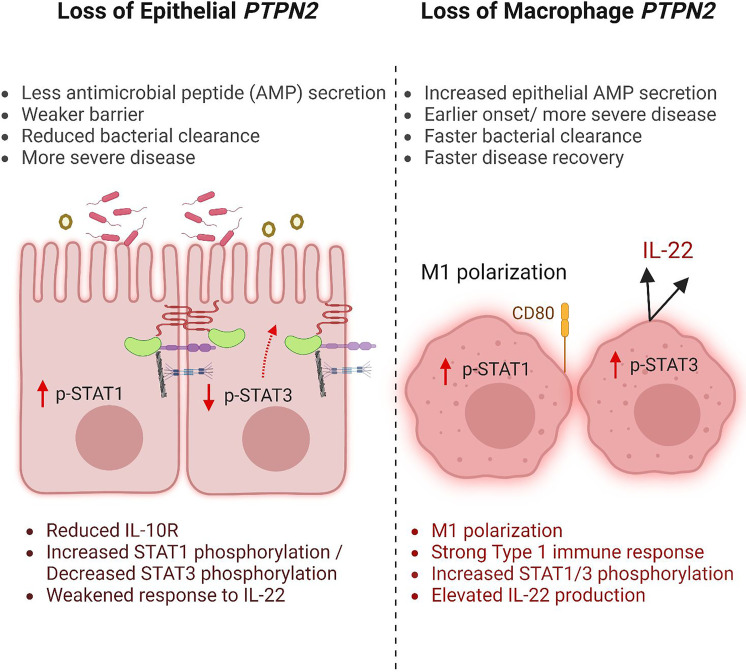

## Introduction

The intestinal epithelium regulates nutrient and fluid and electrolyte uptake, acts as a physical barrier to prevent bacteria from invading the host, and provides important cues to the underlying immune system to maintain tolerance to food antigens and commensal bacteria (1–3). In turn, immune cells influence the function of the epithelial barrier by providing stimuli that promote intestinal epithelial cell (IEC) barrier function and the expression of barrier-sealing molecules (4, 5). During gastrointestinal infections, immune cells in the intestine induce strong immune responses to eliminate invading pathogens (4, 6, 7). These immune responses can have detrimental effects on the epithelium, initiating premature IEC death while also promoting fluid secretion to help eliminate pathogens from the body (8). Besides initiating strong immune responses to eliminate infectious agents, control of the inflammatory response upon clearance of the infection is mandatory to prevent excessive and/or chronic inflammatory responses (4, 6).

Intestinal macrophages, which reside close to IECs, crucially influence the body’s response to invading pathogens by promoting barrier functions of IECs and preventing systemic dissemination of bacteria that breach the epithelial barrier (4, 6). During physiological conditions, intestinal macrophages secrete large amounts of antiinflammatory cytokines and imprint tolerance in other immune cells (6, 9, 10), but rapidly acquire an inflammatory phenotype in the presence of invading pathogens. Consequently, intestinal macrophages are indispensable for mounting efficient protective immune responses (4, 9). Previous studies have investigated how macrophages and other mononuclear phagocytes interact with other immune cells and how they directly react to bacterial products to provide protection against pathogens (6, 11), but it is still poorly understood how macrophage–IEC interactions contribute to combating invading pathogens or how disturbed macrophage–IEC interplay affects the ability of the body to defeat infections.

We recently demonstrated that loss of protein tyrosine phosphatase nonreceptor type 2 (PTPN2) critically alters the interaction of IECs with macrophages and compromises the barrier-promoting effect of intestinal macrophages (5). Variants in *PTPN2* have been associated with several inflammatory disorders (e.g., inflammatory bowel disease, celiac disease, diabetes) (12–14). The role of PTPN2 in T cell functions has been studied in depth, and several studies demonstrated that PTPN2 loss in T cells promotes tissue inflammation, Th1 responses, and aberrant Treg differentiation (15–18). Our group demonstrated that PTPN2-deficient IECs are more susceptible to IFN-γ–induced barrier defects (19–21). Furthermore, full body loss of PTPN2 results in severe systemic inflammation and death within 5 weeks after birth (22, 23). Despite this striking phenotype in homozygous PTPN2-deficient mice, PTPN2-heterozygous (Het) mice do not show signs of spontaneous systemic inflammation, although PTPN2 expression is reduced and these mice have been reported to exhibit a more severe response to chemical colitis models (24). In addition, we recently demonstrated that PTPN2 deficiency in macrophages compromises the barrier functions of IECs, whereas its loss in IECs promotes pro-inflammatory macrophage polarization (5) and promotes IEC barrier defects in response to inflammatory cytokines (5). Thus, PTPN2 deficiency — either heterozygous in the whole body or homozygous tissue-specific deletion in macrophages or IECs — can serve as a model for compromised macrophage–IEC cross-regulation. Although many studies have investigated how disturbed immune functions or a compromised ability of IECs to respond to microbes affect the host’s ability to defeat enteric pathogens, it is not known how compromised IEC–macrophage cross-regulation affects susceptibility and the course of enteric infection.

In this report, we demonstrate that disrupted macrophage–IEC interaction, here modeled by PTPN2 loss in macrophages or IECs, affects the body’s ability to defeat enteric pathogens. We show that PTPN2 loss in macrophages promotes a strong type 1 immune response, which is necessary to promote clearance of and recovery from infection with *Citrobacter rodentium*, a mouse pathogen that models human enteropathogenic and enterohemorrhagic *E*. *coli* infection. Furthermore, we provide evidence that the ability of macrophages to promote antimicrobial defense mechanisms in IECs is dependent on macrophage-intrinsic IL-22 production and that loss of PTPN2 in IECs severely compromises the production of antimicrobial peptides in IECs upon *C*. *rodentium* infection.

## Results

### Lack of PTPN2 in myeloid cells results in more severe early disease but faster clearance of bacteria.

We previously showed that loss of PTPN2 in macrophages affects macrophage–IEC interactions (5). However, to date, it is unclear how PTPN2 loss in different cell types affects the ability to respond to intestinal pathogens. To address how loss of PTPN2 in macrophages or other myeloid cells affects susceptibility to an enteric pathogen, we subjected *Ptpn2*-LysM-Cre mice (25) to *C*. *rodentium* infection.

**Figure 1:**
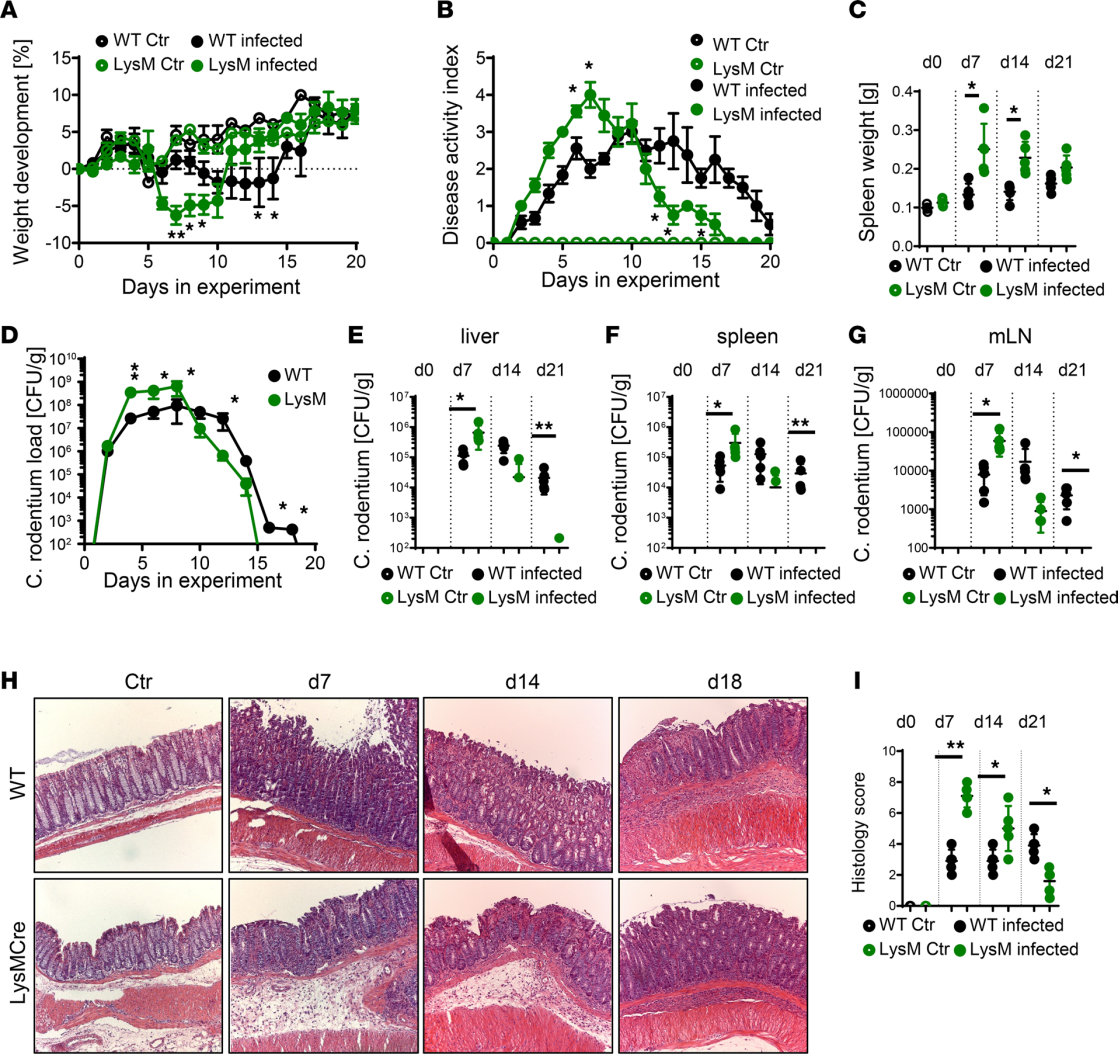
*Ptpn2-*LysM-Cre mice have increased initial disease but clear the infection faster. *PTPN2*-LysM-Cre (LysM) mice, 8–12 weeks old, and their *Ptpn2^fl/fl^* littermates (WT) were infected with 5 × 10^8^ CFU of *C*. *rodentium*. The graphs show (**A**) weight development; (**B**) disease activity; (**C**) spleen weight on indicated days; *C*. *rodentium* load in (**D**) feces over time; and (**E**) liver, (**F**) spleen, and (**G**) mesenteric lymph nodes (mLN) at the indicated days. (**H**) Representative histological pictures of the distal colon at the indicated day after infection. Original magnification, 100×. (**I**) Scoring of damage and infiltration. Representative results from 1 of 2 experiments with *n* = 5/group; *P < 0.05, **P < 0.01 by 2-way ANOVA with Tukey’s post hoc test (**A**, **B**, and **D**) or 2-sided Student’s *t* test (**C**, **E**–**G**, and **I**). Ctr, control; d, day.

As shown previously, in *Ptpn2*-LysM-Cre mice, macrophages are the primary cell type lacking PTPN2 expression, whereas PTPN2 expression is not reduced in other myeloid immune cells such as DCs and granulocytes (26) (Supplemental Figure 1; supplemental material available online with this article; https://doi.org/10.1172/jci.insight.156909DS1). In WT mice, *C*. *rodentium* infection resulted in delayed weight gain or mild weight loss between days 5 and 10, after which they started to recover (Figure 1A). In contrast, *Ptpn2*-LysM-Cre mice had severe, early-onset disease characterized by drastic weight loss, elevated disease activity scores, elevated spleen weight at days 7 and 14, and increased bacterial load in stool, liver, spleen, and in mesenteric lymph nodes early in infection. However, after day 10, *Ptpn2*-LysM-Cre mice started to recover much faster than their *Ptpn2*^fl/fl^ littermates (Figure 1, A–G). This accelerated onset and resolution of disease was also apparent in elevated levels of early histological disease parameters, such as increased infiltration of immune cells and more drastic epithelial hyperplasia (Figure 1, H and I), indicating that although loss of PTPN2 in monocytes or macrophages invoked a more aggressive onset of disease, this was also accompanied by faster clearance of the bacteria.

### Reduced PTPN2 expression results in an enhanced type 1 immune response and elevated IL-22 levels.

To assess why *Ptpn2*-LysM-Cre mice were more susceptible to *C*. *rodentium* infection but, at the same time, able to clear the infection more efficiently, we analyzed the immune cell compartment in these mice over time. This revealed that *Ptpn2*-LysM-Cre mice had elevated proportions of pro-inflammatory (M1) macrophages compared with reduced antiinflammatory (M2) macrophages, and increased levels of IFN-γ^+^ Th cells, thus indicating an overall elevated type 1 immune response (Supplemental Figure 2). Although an elevated type 1 immune response can explain more pronounced weight loss and more severe initial disease, it cannot fully explain faster recovery and bacterial clearance. Thus, we further examined factors that might contribute to bacterial clearance in response to *C*. *rodentium* infection.

**Figure 2:**
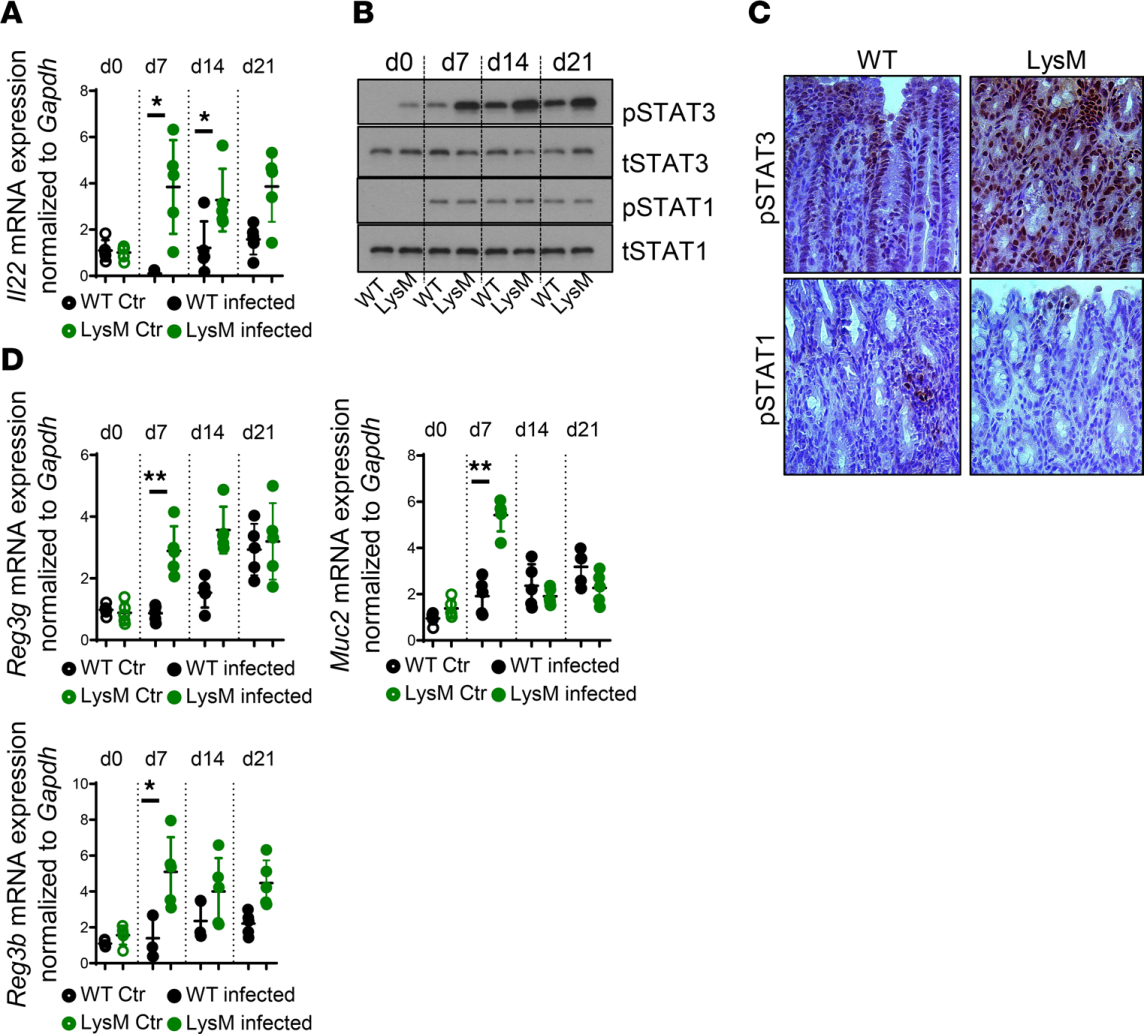
Loss of *Ptpn2* in macrophages promotes IL-22 expression in the inflamed intestine and antimicrobial peptide expression in IECs. *PTPN2*-LysM-Cre (LysM) mice, 8–12 weeks old, and their *Ptpn2^fl/fl^* (WT) littermates were infected with 5 × 10^8^ CFU of *C*. *rodentium*. (**A**) *Il22* mRNA expression in the colon. (**B**) Phospho-STAT3 (pSTAT3) and total STAT3 (tSTAT3) and phospho-STAT1 (pSTAT1) and total STAT1 (tSTAT1) in the colon on day 7 after infection. (**C**) Representative images of pSTAT3 and pSTAT1 in the colon. Original magnification, 200×. (**D**) mRNA expression of *Reg3g*, *Reg3b*, and *Muc2* in the colon. *P < 0.05, **P < 0.01 by 2-sided Student’s *t* test. Representative results from 1 of 2 experiments with *n* = 5/group. Ctr, control; d, day.

It has been reported that IgG-mediated neutrophil activation contributes to bacterial clearance upon *C*. *rodentium* infection (27). However, we did not observe any difference in IgG levels in *Ptpn2*-LysM-Cre mice (Supplemental Figure 3A), ruling out that IgG mediates the accelerated clearance of *C*. *rodentium* in these mice. Furthermore, and in line with minor effects on *Ptpn2* expression in granulocytes in the *Ptpn2*-LysM-Cre mouse (26) (Supplemental Figure 1), granulocytes from *Ptpn2*-LysM-Cre mice showed no difference in their ability to kill *C*. *rodentium* (Supplemental Figure 3) (28). We next analyzed levels of the IL-10 family cytokine IL-22, which has been demonstrated to be crucially involved in protecting the intestinal epithelium from bacterial infections (29, 30) and which can be promoted upon the induction of type 1 immune responses (31). Notably, we detected highly increased mRNA levels of *Il22* in *Ptpn2*-LysM-Cre mice (Figure 2A). In line with elevated IL-22 levels, we also observed increased levels of STAT3 (Y^705^) phosphorylation in IECs of *Ptpn2*-LysM-Cre mice (Figure 2, B and C). *Ptpn2*-LysM-Cre mice had an elevated type 1 immune response, indicating elevated presence of STAT1-inducing cytokines. However, STAT1 phosphorylation (Y^701^) levels were not elevated in IECs from *Ptpn2*-LysM-Cre mice, which is likely the result of intact PTPN2 function and subsequently normal STAT1 dephosphorylation in these cells (Figure 2, B and C). In *Ptpn2*-LysM-Cre mice, elevated STAT3 levels in IECs were paralleled by an increase in mRNA expression of antimicrobial factors such as regenerating islet-derived protein 3 gamma and beta (*Reg3g*, *Reg3b*) and *Muc2* in IECs (Figure 2D). This finding indicates that elevated production of barrier-protecting factors seems to contribute to faster clearance of *C*. *rodentium* infection in *Ptpn2-*LysM-Cre mice.

### PTPN2-deficient macrophages express highly elevated levels of IL-22.

**Figure 3:**
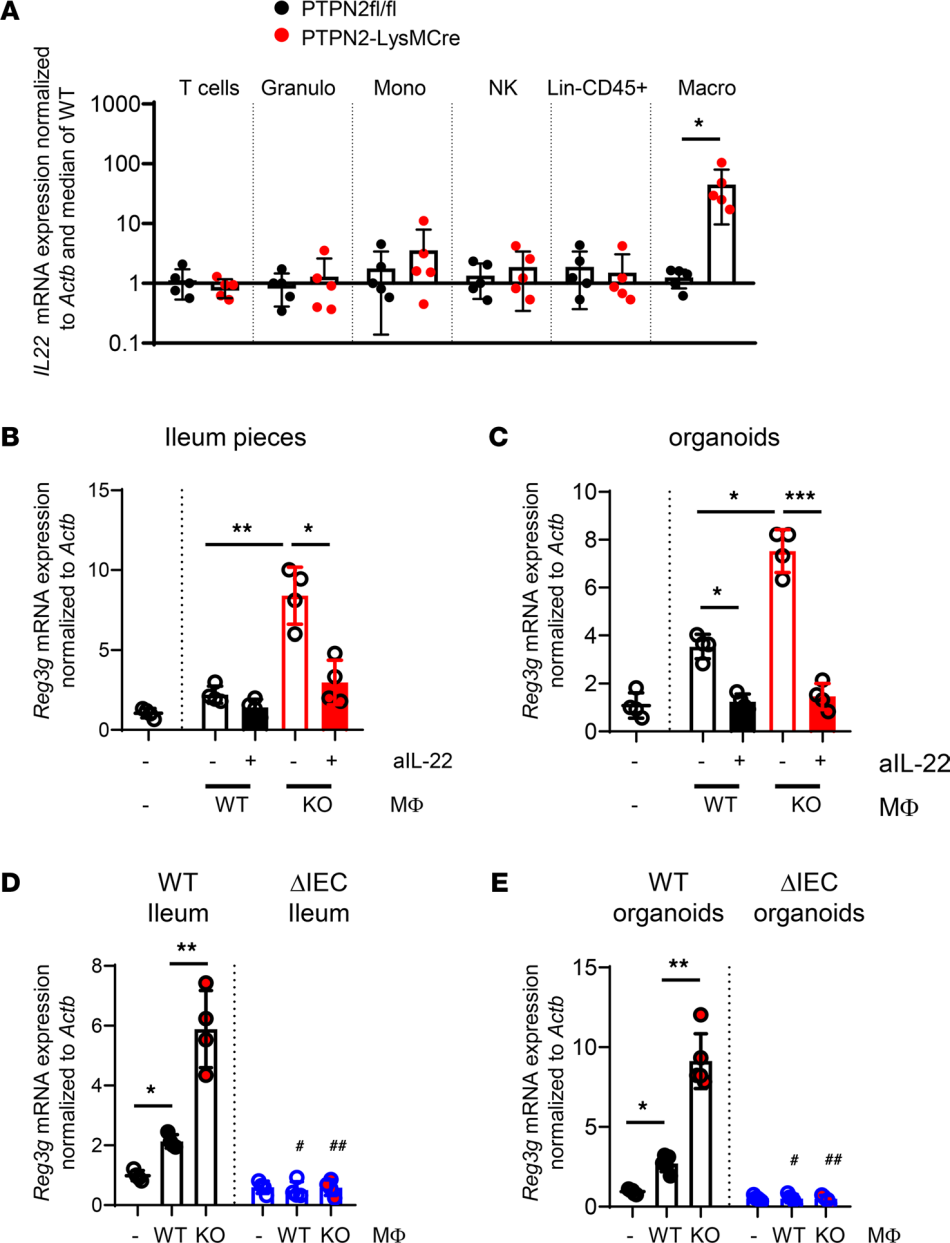
*Ptpn2-*deficient macrophages promote *Reg3g* expression in an IL-22–dependent manner. (**A**) T cells (live, CD45^+^, CD3^+^ cells); granulocytes (granulo) (live, CD45^+^, Ly6G^+^ cells); monocytes (mono) (live, Cd45^+^, CD11b^hi^, Ly6C^+^, MHCII^–^ cells); NK cells (live, CD45^+^, NK1.1^+^, CD3^–^ cells); lineage-negative, CD45^+^ (lin^–^CD45^+^) cells (live, CD3-B220^–^, NK1.1^–^, CD11b^–^, CD11c^–^, Ly6C^–^, Ly6G^–^, F4/80^–^, CD64^–^ cells); and macrophages (macro) (live, CD45^+^, Cd11b^+^, CD64^+^, F4/80^+^ cells) were sorted from the spleen of *Ptpn2^fl/fl^* and *Ptpn2-*LysM-Cre mice and analyzed for *Il22* mRNA expression. (**B**) Small intestinal explants from WT mice or (**C**) small intestinal organoids from WT mice were cultured for 24 hours with bone marrow macrophages from *Ptpn2^fl/fl^* mice (WT) and *Ptpn2-*LysM-Cre littermates (KO) in presence of an isotype control (negative) or an anti–IL-22 (^+^) neutralizing Ab. (**D**) Small intestinal explants from *Ptpn2*^ΔIEC/ERT^ (ΔIEC/ERT) or *Ptpn2^fl/fl^* littermates (fl/fl) were cultured for 24 hours with bone marrow macrophages from *Ptpn2^fl/fl^* mice (WT) and *Ptpn2-*LysM-Cre littermates (KO). (**E**) Organoids derived from the small intestine of naive *Ptpn2*^ΔIEC/ERT^ (ΔIEC/ERT) or *Ptpn2^fl/fl^* littermates (WT) were exposed to 4-hydroxytamoxifen for 48 hours to induce recombination. Tamoxifen was removed and organoids subcultured for 3 additional days before coculture with bone marrow macrophages from *Ptpn2^fl/fl^* mice (WT) and *Ptpn2-*LysM-Cre littermates (KO) for 24 hours. *P < 0.05, **P < 0.01, ***P < 0.001; ^#^*P* < 0.05 compared with WT ileum with WT macrophages, ^##^*P* < 0.01 compared with WT ileum with KO macrophages; 2-sided *t* test (**A**) or 1-way ANOVA (**B**–**E**). Each dot represents an independent biological replicate, *n* = 5 (**A**) or *n* = 4 (**B**–**E**). Δ, change in; Mφ, macrophage.

To investigate which cells were responsible for the elevated production of IL-22 in *Ptpn2*-LysM-Cre mice, we sorted T cells, granulocytes, monocytes, innate lymphoid cells, and macrophages from the spleen. Except for macrophages, all of these cells expressed normal levels of *Il22* upon deletion of *Ptpn2* in macrophages (Figure 3A). This finding indicates that the increased levels of IL-22 observed in *Ptpn2-*LysM-Cre mice within the colon of *C*. *rodentium*–infected *Ptpn2*-LysM-Cre mice originated from macrophage-intrinsic, elevated IL-22 levels rather than the induction of IL-22 in other cells. This was verified at the protein level, where we found that in WT mice, macrophages were responsible for approximately 5% of all IL-22 secreted from lamina propria immune cells, whereas in *Ptpn2*-LysM-Cre mice, macrophages accounted for 40%–60% of total IL-22 production (Supplemental Figure 4A). Notably, *Ptpn2*-deficient granulocytes (isolated from *Ptpn2*-KO mice) produced similar levels of IL-22 as their WT counterparts (Supplemental Figure 4B). Thus, PTPN2-deficient macrophages might be directly responsible for driving enhanced expression of antibacterial defense molecules in IECs, although a more detailed assessment of *Ptpn2* deletion on neutrophil function and/or IL-22 production and the consequences for *C*. *rodentium* clearance will require additional, more extensive studies.

Culturing colon explants as well as intestinal organoids from WT mice with PTPN2-deficient macrophages resulted in elevated levels of *Reg3g* expression in an IL-22–dependent manner (Figure 3, B and C). Notably, *Ptpn2*-deficient macrophages were unable to induce *Reg3g* expression in cocultured colon explants from mice lacking *Ptpn2* in IECs (*Ptpn2*-VilCreERT mice; hereafter, *Ptpn2*^ΔIEC^ mice) (Figure 3D and Supplemental Figure 5A) or in organoids derived from those mice (Figure 3E). Notably, culture of organoids or colon explants with T cells or granulocytes from *Ptpn2^–/–^* mice or macrophage-depleted, CD45^+^ spleen cells from *Ptpn2*-LysM-Cre mice did not result in elevated *Reg3g* expression when compared with cocultures with the respective cells from *Ptpn2^fl/fl^* control mice (Supplemental Figure 5B). This finding strongly indicates that loss of *Ptpn2* promotes IL-22 secretion from macrophages, which enhances antibacterial defense and thus faster clearance of the infection. However, this mechanism seems to be disturbed when epithelial cells lack *Ptpn2*.

### Mice lacking intestinal epithelial Ptpn2 suffer from enhanced C. rodentium infection and show delayed clearance of the infection.

**Figure 4:**
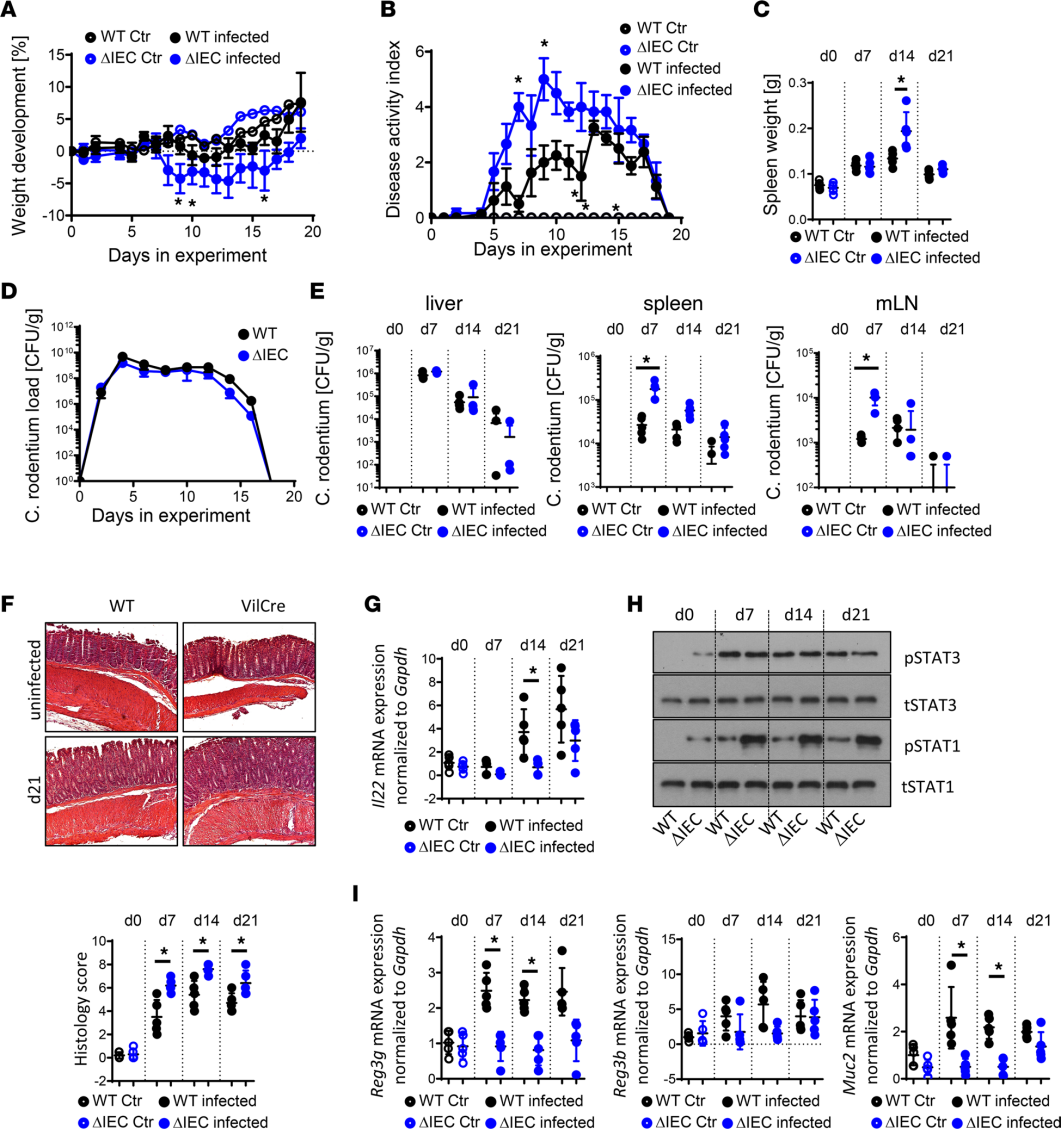
*Ptpn2*^ΔIEC/ERT^ mice have pronounced *C*. *rodentium*–induced disease. *Ptpn2*^ΔIEC/ERT^ (ΔIEC/ERT) mice (5–8 weeks old) and their *Ptpn2^fl/fl^* littermates (WT) were injected with 1 mg/kg tamoxifen for 5 consecutive days. Four weeks later, the mice were infected with 5 × 10^8^ CFU of *C*. *rodentium*. The graphs show (**A**) weight development, (**B**) disease activity, and (**C**) spleen weight on indicated days; *C*. *rodentium* load in (**D**) feces over time and in (**E**) liver, spleen, and mesenteric lymph nodes (mLN) at the indicated days. (**F**) Representative histological pictures of the distal colon at the indicated day after infection and scoring of damage and infiltration. Original magnification, 100×. (**G**) *Il22* mRNA expression. (**H**) Phospho-STAT3 (pSTAT3) and total STAT3 (tSTAT3) and phospho-STAT1 (pSTAT1) and total STAT1 (tSTAT1) in the colon. (**I**) *Reg3g*, *Reg3b*, and *Muc2* mRNA expression in the colon. Representative results from 1 of 2 experiments with *n* = 3–5/group; each dot represents 1 mouse. *P < 0.05 by 2-way ANOVA (**A**, **B**, and **D**) or Student’s 2-sided *t* test (**C**, **E**–**G**, and **I**). Δ, change in; Ctr, control; d, day.

Because we observed that *Ptpn2* in IECs is required for the induction of antimicrobial peptides by *Ptpn2-*deficient macrophages, we further examined how loss of *Ptpn2* in IECs affects susceptibility to *C*. *rodentium* infection and disease. Here, we used *Ptpn2*^ΔIEC^ mice in which PTPN2 was specifically deleted in all IECs upon tamoxifen injection (Supplemental Figure 6). Four weeks after tamoxifen injection, *Ptpn2*^ΔIEC^ mice were infected with *C*. *rodentium*, which resulted in delayed weight gain and elevated disease activity when compared with *Ptpn2^fl/fl^* littermates. Despite elevated disease and increased bacterial load, *Ptpn2*^ΔIEC^ mice ultimately were able to clear the infection (Figure 4, A–F). Of interest, and in contrast to LysM-Cre mice, *Ptpn2*^ΔIEC^ mice showed no increase in type 1 immune responses (Supplemental Figure 7) and no increase in IL-22 levels (Figure 4G).

In line with PTPN2 being a negative regulator of STAT1 and STAT3, we observed increased levels of STAT1 and STAT3 phosphorylation in isolated IECs from naive (day 0) *Ptpn2*^ΔIEC^ mice (Figure 4H) and clearly increased levels of STAT1 upon *C*. *rodentium* infection. However, *Ptpn2*^ΔIEC^ mice did not induce STAT3 activation in IECs or increase expression of antimicrobial defense molecules after infection (Figure 4, H and I), despite normal expression of *Il22r1* and *Il10r2* (Supplemental Figure 5, C and D). This observation indicates that deletion of PTPN2 renders IECs incapable of inducing factors that are involved in clearing intestinal pathogens. Notably, although resulting in reduced disease and accelerated recovery from disease in WT littermates, treatment with recombinant IL-22 did not reduce disease severity or promote recovery in *Ptpn2*^ΔIEC^ mice (Supplemental Figure 8, A and B), indicating that the more severe disease in these mice was not due to reduced IL-22 induction but more likely was due to IEC-intrinsic defects in production of antimicrobial peptides that could not be rescued by in vivo treatment with IL-22 (Supplemental Figure 8, C and D).

### Ptpn2-deficient macrophages are responsible for the faster clearance of the infection in Ptpn2-LysM-Cre mice.

**Figure 5:**
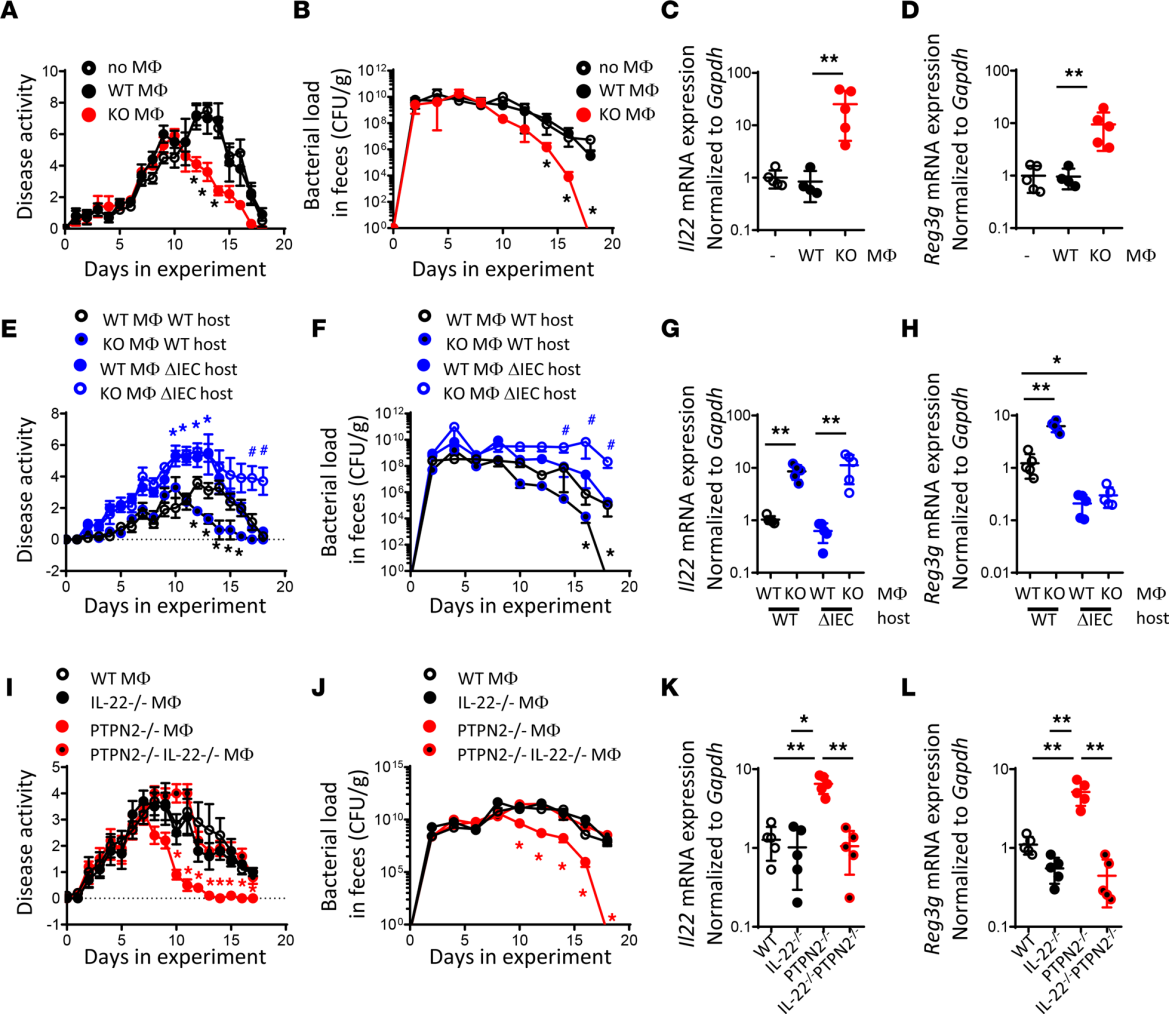
IL-22 expression in *Ptpn2-*deficient macrophages is necessary to promote faster recovery. (**A–D**) WT mice were infected with 5 × 10^8^ CFU of *C*. *rodentium*. On day 7, bone marrow–derived macrophages from *Ptpn2^fl/fl^* mice (WT) and *Ptpn2-*LysM-Cre littermates (KO) were transferred via i.p. injections. (**E**–**H**) *Ptpn2*^ΔIEC/ERT^ (ΔIEC/ERT2) mice (5–8 weeks old) and their *Ptpn2^fl/fl^* littermates (WT) were injected with 1 mg/kg tamoxifen for 5 consecutive days. Four weeks later, the mice were infected with 5 × 10^8^ CFU of *C*. *rodentium*. On day 7 after infection, bone marrow–derived macrophages from *Ptpn2^fl/fl^* mice (WT) and *Ptpn2-*LysM-Cre littermates (KO) were transferred via i.p. injections. (**I**–**L**) WT mice were infected with 5 × 10^8^ CFU of *C*. *rodentium*. On day 7, bone marrow–derived macrophages from *Ptpn2^fl/fl^* mice (WT), *Il22-*KO (IL22^–/–^), *Ptpn2-*LysM-Cre (PTPN2^–/–^), or *Il22-*KO-*Ptpn2-*LysM-Cre (IL-22^–/–^PTPN2^–/–^) mice were transferred via i.p. injections. Depicted are (**A**, **E**, and **I**) weight development, (**B**, **F**, and **J**) CFU of *C*. *rodentium* in the feces over time, (**C**, **G**, and **K**) *Il22* mRNA expression in the colon, and (**D**, **H**, and **L**) *Reg3g* mRNA expression in the colon. Each dot represents an individual mouse; *n* = 5 per group. *P < 0.05, **P < 0.01; ^#^*P* < 0.05 KO Mφ ΔIEC host compared with WT Mφ ΔIEC host by 2-way ANOVA (**A**, **B**, **E**, **F**, **I**, and **J**) or 1-way ANOVA (**C**, **D**, **G**, **H**, **K**, and **L**). Δ, change in; Mφ, macrophage.

Having shown that loss of PTPN2 in macrophages results in elevated IL-22 levels and the promotion of antimicrobial peptides, we next aimed to investigate whether *Ptpn2*-deficient macrophages indeed are responsible for the faster disease clearance in *Ptpn2*-LysM-Cre mice. To address this, we generated bone marrow–derived macrophages from WT or *Ptpn2*-LysM-Cre mice and transferred them into *C*. *rodentium*–infected WT mice. *Ptpn2*-deficient macrophages were able to promote bacterial clearance, and mice receiving *Ptpn2-*deficient macrophages had elevated IL-22 levels in the colon and increased *Reg3g* mRNA expression in IECs (Figure 5, A–D), demonstrating that *Ptpn2*-deficient macrophages are sufficient to promote disease recovery.

Nevertheless, when transferred into *Ptpn2*^ΔIEC^ mice, *Ptpn2*-deficient macrophages not only were unable to promote recovery but also further worsened the infection, resulting in a severe delay in recovery and a failure to clear bacterial load (Figure 5, E–H). This finding not only indicates that macrophages from *Ptpn2*-LysM-Cre mice promote bacterial clearance, possibly via increased IL-22 secretion, but also demonstrates that *Ptpn2*-deficient IECs are incapable of responding adequately to these disease-alleviating cues and that, upon deletion of *PTPN2* in IECs, *Ptpn2*-deficient macrophages lose their capacity to alleviate disease and instead worsen the disease outcomes of infection.

### IL-22 expression in Ptpn2-deficient macrophages is essential to promote recovery.

To investigate whether macrophage-derived IL-22 is the critical factor that promotes recovery from *C*. *rodentium* infection upon transfer of *Ptpn2*-deficient macrophages, we next transferred *Ptpn2*-deficient macrophages that expressed IL-22 normally, or *Ptpn2*-deficient macrophages in which the *Il22* gene was disrupted, into *C*. *rodentium*–infected hosts. As observed by a lack of faster recovery, failure to induce accelerated bacterial clearance, and the absence of enhanced *Reg3g* mRNA induction, disruption of the *Il22* gene in *Ptpn2*-deficient macrophages negated the beneficial effect of these cells (Figure 5, I–L). These data strongly indicate that *Ptpn2*-deficient macrophages require IL-22 to mediate a beneficial, disease-alleviating effect in vivo.

### Ptpn2-heterozygous mice are more susceptible to C. rodentium infection.

In humans carrying disease-associated *PTPN2* variants, PTPN2 is not completely deleted, but its function is reduced (5). Furthermore, PTPN2 dysfunction affects all cells of the body, not only macrophages or IECs. Thus, to more closely reflect the situation in human patients, we subjected *Ptpn2-*Het mice 8–12 weeks old (similar to humans carrying *PTPN2* variants, these mice show reduced, but not absent, PTPN2 activity in all cells of the body) to *C*. *rodentium* infection. In line with more severe early disease in *Ptpn2-*LysM-Cre mice and delayed recovery in *Ptpn2*^ΔIEC^ mice, *Ptpn2-*Het mice had more severe disease throughout the experiment, as evidenced by reduced weight gain, elevated disease activity, larger spleens, and elevated histological scores (Supplemental Figure 9). Similar to *Ptpn2-*LysM-Cre mice, *Ptpn2-*Het mice had increased type I immune response (Supplemental Figure 10) and elevated STAT3 phosphorylation in IECs (Supplemental Figure 11, A–C). However, like *Ptpn2* ^ΔIEC^ mice, *Ptpn2-*Het mice with elevated STAT1 phosphorylation did not induce antimicrobial peptides (Supplemental Figure 11). Overall, *Ptpn2-*Het mice did not recover from the infection and had a similar phenotype to *Ptpn2* ^ΔIEC^ mice receiving transferred PTPN2-deficient macrophages (compare Figure 5, E–H). Taken together, these findings indicate that not only homozygous (thus complete) loss of PTPN2 in macrophages and IEC renders the host prone to more severe disease upon infection with enteropathogens, but partial loss of PTPN2 — akin to patients carrying *PTPN2* loss of function variants — also renders the host unable to adequately respond to the infection.

## Discussion

We demonstrate that disturbed macrophage–IEC interactions, caused by PTPN2 deletion in either macrophages or IECs, has detrimental consequences for the host’s ability to defeat enteric pathogens and that PTPN2 loss promotes susceptibility to infection with *C*. *rodentium*, a model organism of enterohemorrhagic *E*. *coli* infections in humans. Our data reveal that PTPN2-deficient macrophages exacerbate type 1 immune responses and production of IL-22 in response to *C*. *rodentium*, resulting in earlier onset and increased severity of disease. Notably, in normal IECs, this elevated type 1 response, and especially the increased production of IL-22 in macrophages, also promoted antimicrobial defense mechanisms and mediated more efficient pathogen clearance. However, PTPN2-deficient IECs were unable to react to these barrier-protecting cues and failed to induce antimicrobial factors in response to *C*. *rodentium* infection. As a consequence, combined loss of PTPN2 in IECs and macrophages resulted in devastating disease and an inability to control the infection efficiently. Similarly, partial loss of whole-body PTPN2 resulted in a failure to clear *C*. *rodentium* infection. These findings show that reduced PTPN2 activity has profound effects on intestinal health and compromises macrophage–IEC interactions during intestinal infection, prompting devastating disease and an inability to prevent bacterial infection.

In this study, we used complementary mouse models to delineate the role(s) of PTPN2 in macrophages (LysM-Cre model) versus IECs (Villin-Cre model). In addition, we used mice with full-body heterozygous deletion of *Ptpn2*, a model that is analogous to the situation in human patients who carry PTPN2 loss-of-function variants. We chose to investigate cell-specific deletion on a PTPN2-sufficient background; however, future studies could investigate cell-specific deletion on a heterozygote background, which might further elucidate the effect of partial loss of PTPN2 on specific cell functions.

Along with enhanced type 1 immune responses, PTPN2 deficiency resulted in drastically elevated production of IL-22, specifically in macrophages. So far, lymphocytes, including T cells and innate lymphoid cells, have been considered the main sources of IL-22 in the intestine (32, 33), whereas secretion from myeloid immune cells has been described but is believed to be less important (33–35). In particular, our macrophage transfer experiments clearly demonstrated that, at least in the setting of PTPN2 deficiency, macrophage-derived IL-22 was necessary to promote epithelial barrier integrity and was required for faster recovery mediated by PTPN2-deficient macrophages. Although additional factors secreted from PTPN2-deficient macrophages (e.g., IL-6, IL-12, IL-23) might contribute to this phenotype (36), our in vitro experiments inhibiting IL-22 and in vivo transfer of IL-22/PTPN2 double-KO macrophages clearly demonstrate that the ability of macrophages to produce IL-22 is required for the protective effect.

Our results demonstrate that macrophage-derived IL-22 is necessary to induce AMP production by IECs; myeloid cell–specific loss of PTPN2 promoted macrophage secretion of IL-22, whereas loss of PTPN2 in IECs rendered the epithelium unresponsive to the barrier-protecting effects of IL-22. Notably, PTPN2-deficient IECs express normal levels of the IL-22 receptor, thus the inability to adequately respond to IL-22 is not due to a failure of IL-22 sensing but rather due to an altered intracellular response upon IL-22 receptor ligation. This is also evidenced by the fact that PTPN2-deficient IECs exhibited elevated levels of STAT1, whereas STAT3 induction was reduced. This is in line with reports showing that exacerbated STAT1 activation can mediate barrier disruption, whereas STAT3 activity downstream of the IL-22 receptor promotes the production and secretion of antimicrobial factors in IECs (33, 37). Thus, an adequate epithelial response to IL-22 is important for its barrier-protective effects, whereas elevated STAT1 activation in response to IL-22 upon PTPN2 loss interrupts the beneficial and barrier-protecting effects.

In summary, our results demonstrate that loss of PTPN2, and subsequently impaired interaction between macrophages and IECs, culminate in an inability to defeat invading pathogens and render the host susceptible to intestinal infections. Our findings not only demonstrate a potentially novel aspect of how loss of PTPN2 compromises intestinal health but also unravel the importance of macrophage-mediated factors and especially macrophage-derived IL-22 for the induction of protective molecules in the intestinal epithelium. Subclinical or underlying intestinal barrier defects not only associate with onset of inflammatory bowel disease but also associate with other chronic inflammatory diseases, such as rheumatoid arthritis and diabetes (38, 39), and gastrointestinal infections have been suggested to trigger or accelerate the development of a number of chronic inflammatory disorders (40, 41). Notably, loss-of-function PTPN2 variants are associated with increased risk of chronic intestinal inflammatory disease, and our findings in *Ptpn2*-Het mice, which exhibit a partial reduction in PTPN2, are consistent with this (14). Thus, identifying how PTPN2 dysfunction affects macrophage–IEC interactions, and the subsequent response of the host to intestinal pathogens, not only are important for understanding downstream mechanisms of intestinal infections but also might help identify individuals at risk for development of chronic gastrointestinal diseases after intestinal infections.

## Methods

### Mice.

*Ptpn2-*LysM-Cre mice were initially obtained from Michael Scharl (University Hospital Zurich, Zurich, Switzerland), and a local colony was maintained by crossing *Ptpn2^fl/fl^* mice with *Ptpn2-*LysM-Cre mice. PTPN2^ΔIEC^ mice were generated by crossing *Ptpn2^fl/fl^* mice with mice expressing the CreERT2 construct under the Villin promoter. For in vivo induction of Cre expression and deletion of PTPN2 in IECs, these mice were injected on 5 consecutive days with 1 mg/kg tamoxifen in 100 μL of corn oil. Experiments were started 4 weeks after the last tamoxifen injection. To generate mice lacking IL-22 and PTPN2 in macrophages, *Ptpn2*-LysM-Cre mice were crossed with IL22-Cre mice (The Jackson Laboratory, catalog 027524). These mice were used as bone marrow donors for bone marrow–derived macrophage (BMDM) generation. *Ptpn2-*heterozygous mice on the BALB/c background were initially obtained from Michel Tremblay (McGill University, Montréal, Québec, Canada) and bred with WT BALB/c mice to obtain WT and *Ptpn2-*heterozygous littermates for experiments.

### C. rodentium infection and monitoring of disease.

Mice were infected by oral gavage with 10^8^–10^9^ CFU of *C*. *rodentium*. Weight development and disease activity (i.e., general appearance, weight development, stool consistency, blood in stool) was monitored daily, as described previously (5). Bacterial burden in feces was determined every second day by homogenizing stool samples in PBS, plating serial dilutions on MacConkey agar plates, and counting colonies after 16 hours of incubation at 37°C. Mice were sacrificed on days 7, 14, and 18–21 after infection. To determine bacterial burden in spleen and mesenteric lymph nodes, the organs were collected, then homogenized in PBS, and serial dilutions were plated on MacConkey agar plates as described above. To assess inflammation, distal colon sections were embedded in formalin, cut into 5 μm sections, stained with H&E, and then scored as described previously (5).

For experiments with IL-22 supplementation, mice were injected twice daily i.p. with recombinant murine IL-22 (2 μg/mouse/injection) or saline control starting on day 3 after infection with *C*. *rodentium*.

### Flow cytometry.

Cells from the lamina propria, lymph node, and spleen were isolated as described previously (15, 42, 43). The cells were then incubated with Fc receptor–blocking Ab (Miltenyi Biotec) for 10 minutes and stained with appropriate Abs: anti–CD45-Pacific Blue, clone 30-F11; anti–CD3-BV650, clone 17A2; anti–NK1.1-BV650, clone PK136; anti–B220-BV650, clone RA3-6B2; anti–CD11b-BV605, clone M1/70; anti–CD11c-PECy7, clone N418; anti–Ly6C-PerCPCy5.5, clone HK1.4; anti–F4/80-Fitc, clone BM8; anti–CD64-PE, clone X54-5; anti–MHC-II-AF700, clone M5/114.15.2; anti–CD206-PE-Texas Red, clone 15-2; FoxP3-APC, clone 206D; anti–IFNg-PE-Cy7, clone XMG1.2; and anti–IL-17-APC, clone TC11-18H10.1 (all from BioLegend) for 15–30 minutes. Zombie NIR live/dead stain (BioLegend) was used for discrimination between live and dead cells in all experiments. For intracellular staining, cells were permeabilized using the FoxP3 staining kit from eBioscience. Samples were acquired on an LSRII cytometer (BD) and analyzed using FlowJo (Tree Star, Inc.). For analysis of PTPN2 expression in different immune cell subsets, the cells were isolated as described above and sorted using a FACSAria III cell sorter from BD, as described before (25, 26).

### Western blotting, quantitative PCR, H&E staining, and IHC.

Protein and RNA isolation, Western blotting, quantitative PCR, and IHC were performed according to standard procedures and as described previously (5). For Western blotting, proteins were isolated using RIPA buffer (150 mM NaCl, 5 mM EDTA, 50 mM Tris, 1% NP-40, 0.5% sodium deoxycholate, and 0.1% SDS, supplemented with complete mini protease inhibitor cocktail from Roche); equal amounts of protein were separated by PAGE and blotted onto PVDF membranes. After blocking with 3% milk and 1% BSA in wash buffer (Tris-buffered saline, 0.1% Tween), the membranes were incubated overnight with primary Abs (see ref. 5 for details on the Abs used), washed, and incubated with HRP-coupled anti–mouse, anti–rabbit, or anti–goat secondary Abs (Jackson ImmunoResearch) prior to detection of immunoreactive proteins using an ECL detection kit (Thermo Fisher Scientific) and x-ray films (Labscientific Inc.). Densitometric analyses were performed using FIJI (ImageJ, NIH). H&E staining and IHC were performed on paraffin-embedded pieces from the distal colon, as described previously (5).

RNA was extracted using the RNeasy Mini Kit from QIAGEN according to the manufacturer’s instructions. Complementary DNA was generated using the qScript cDNA synthesis kit from Quantabio and iQ SYBR Green Supermix (Bio-Rad) used for real-time quantitative PCR performed on a C1000 Thermal cycler equipped with a CFX96 Real-Time PCR system and the BioRad CFX Manager 3.1 software. Primers were the same as described before (5).

### Intestinal explants.

Small intestinal pieces, 0.5 cm long, were opened longitudinally, then washed thoroughly in PBS containing 1× penicillin/streptomycin (Pen/Strep; Thermo Fisher Scientific) prior to incubation in DMEM supplemented with 10% FCS and 1× Pen/Strep solution. In coculture experiments, 500 macrophages were seeded onto the well, allowed to adhere for 6 hours, and washed with PBS prior to addition of DMEM supplemented with 10% FCS and 1× Pen/Strep solution and addition of intestinal explants. For experiments with IL-22 inhibition, 100 ng/mL anti–IL-22 (PeptroTech) was added to the culture medium during cocultures. RNA was isolated after 24 hours.

### Intestinal organoids.

Crypts were isolated from WT mice, and organoids were generated as described previously (5). We seeded 600 organoids per cell culture insert, and these were maintained in mouse minimal medium. After 3 days, the organoid-derived monolayer containing inserts were transferred into wells containing 500,000 macrophages and cultured for 24 hours prior to collection of RNA.

### BMDMs and in vivo macrophage transfer.

BMDMs were generated as previously described (5). On day 7 of culture, the cells were collected using 2 mM EDTA in PBS, then washed 3 times in ice-cold PBS, and 3 × 10^6^ live macrophages were injected in the tail vein of recipient mice on day 8 after *C*. *rodentium* infection. Macrophage identity was confirmed by flow cytometry on the basis of CD64 and F4/80 expression and viability assessed using Zombie NIR staining. Greater than 97% of the cells were CD64 and F4/80 double positive and viability was typically approximately 85%.

### Analyses of IgG levels in serum and colonic tissue.

IgG levels were analyzed using a commercially available IgG ELISA against all IgG isotypes. For analysis in serum samples, samples were diluted 1:10,000. For analysis in colon tissue, colon tissues were mechanically homogenized in PBS using a GentleMACS tissue dissociator (Miltenyi Biotec).

### Isolation of granulocytes and analysis of bacterial killing.

Granulocytes were isolated from bone marrow as described previously (5) via discontinuous density gradient centrifugation using sterile and endotoxin-free reagents and material. Purity of granulocytes was confirmed by flow cytometry and found to be higher than 90% in all samples. For bacterial killing assessment, granulocytes were incubated with *C*. *rodentium* at an MOI of 10, as described previously (28, 44). At 30 minutes after infection, the cells were washed twice with gentamycin-containing medium, and bacterial load was determined 3 hours later.

### Analysis of IL-22 production.

To determine the amount of IL-22 secreted by macrophages, lamina propria mononuclear cells (LPMCs) were isolated as described (5). Macrophages were then removed from half of the obtained LPMCs, using a macrophage isolation kit from Miltenyi Biotec. LPMCs or macrophage-depleted LPMCs were then incubated overnight in RPMI supplemented with 5% FCS, and the supernatant was analyzed by ELISA using a commercially available IL-22 kit (R&D Systems). For measurement of IL-22 production from granulocytes, granulocytes were isolated as described above and incubated overnight in RPMI 2% FCS prior to analysis of IL-22 in the supernatant, using an IL-22 ELISA kit from R&D Systems.

### Statistics.

Data are presented as mean ± SD of the number of biological replicates. Animal data are presented as mean ± SD and represent pooled data from 2 independent experiments, each with 3–6 animals per group. Statistical analysis was performed using 1-way ANOVA followed by Dunnett’s test, Holm-Šídák posttest, or Kruskal-Wallis test (endpoint measurements) or by 2-way ANOVA (time series measurements). *P* < 0.05 was considered significant. Statistical analysis was performed using GraphPad Prism, version 8 (GraphPad Software).

### Study approval.

The local IACUC at University of California, Riverside, approved all animal experiments.

## Author contributions

MRS conceived the study, contributed to the study design and writing of the manuscript, performed experiments, and analyzed and interpreted the data. VC, AB, AS MC, ANS, PC, and JL performed experiments and analyzed and interpreted the data. MGN contributed to data interpretation and the study design and provided critical intellectual input. DFM conceived the study, contributed to data interpretation and writing of the manuscript, supervised experiments, and acquired funding. All authors reviewed and edited the manuscript.

## Supplementary Material

Supplemental data

## Version 1 02/22/2023

Electronic publication
