# Supplementary material for: PTPN2 regulates bacterial clearance in a mouse model of enteropathogenic and enterohemorrhagic E. coli infection
Source: JCI Insight. 2023 Feb 22;8(4):e156909. doi: 10.1172/jci.insight.156909 (PMC9977497; doi:10.1172/jci.insight.156909)
Supplement: Supplemental data [file jciinsight-8-156909-s006.pdf]

Supplementary Files to:

PTPN2 regulates bacterial clearance in a mouse model of enteropathogenic and enterohemorrhagic *E. coli* infection

Marianne R. Spalinger[1,2], Vinicius Canale[1], Anica Becerra[1], Ali Shawki[1], Meli'sa Crawford[1], Alina N. Santos[1], Pritha Chatterjee[1], Jiang Li[1], Meera G. Nair[1], Declan F. McCole[1]

[1]*Division of Biomedical Sciences, School of Medicine, University of California Riverside, Riverside, California;* [2]*Department for Gastroenterology and Hepatology, University Hospital Zurich and University of Zurich, Zurich, Switzerland*

FIGURE S1

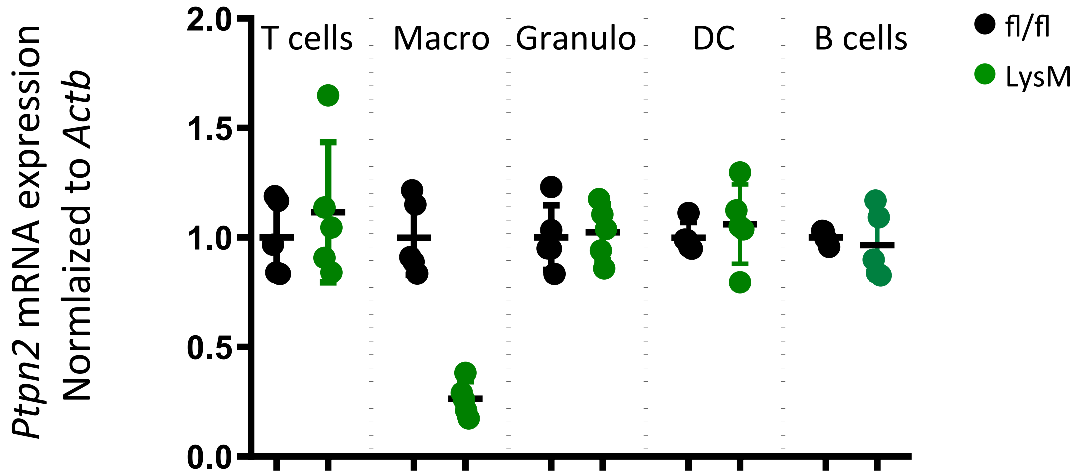

Supplementary figure S1. In PTPN2-LysMCre mice, PTPN2 expression is mainly reduced in macrophages and monocytes but not in granulocytes. 8-12 week old *PTPN2*-LysMCre (LysM) mice and their *Ptpn2*$^{fl/fl}$ littermates (fl/fl) were infected with 5x10^8 CFU *C. rodentium.* Colonic lamina propria cells were isolated 7 days post infection, and the indicated immune cell population sorted to analyze *Ptpn2* mRNA expression. Each dot represents values from an individual mouse, ** = p<0.01. Related to main figure 1

FIGURE S2

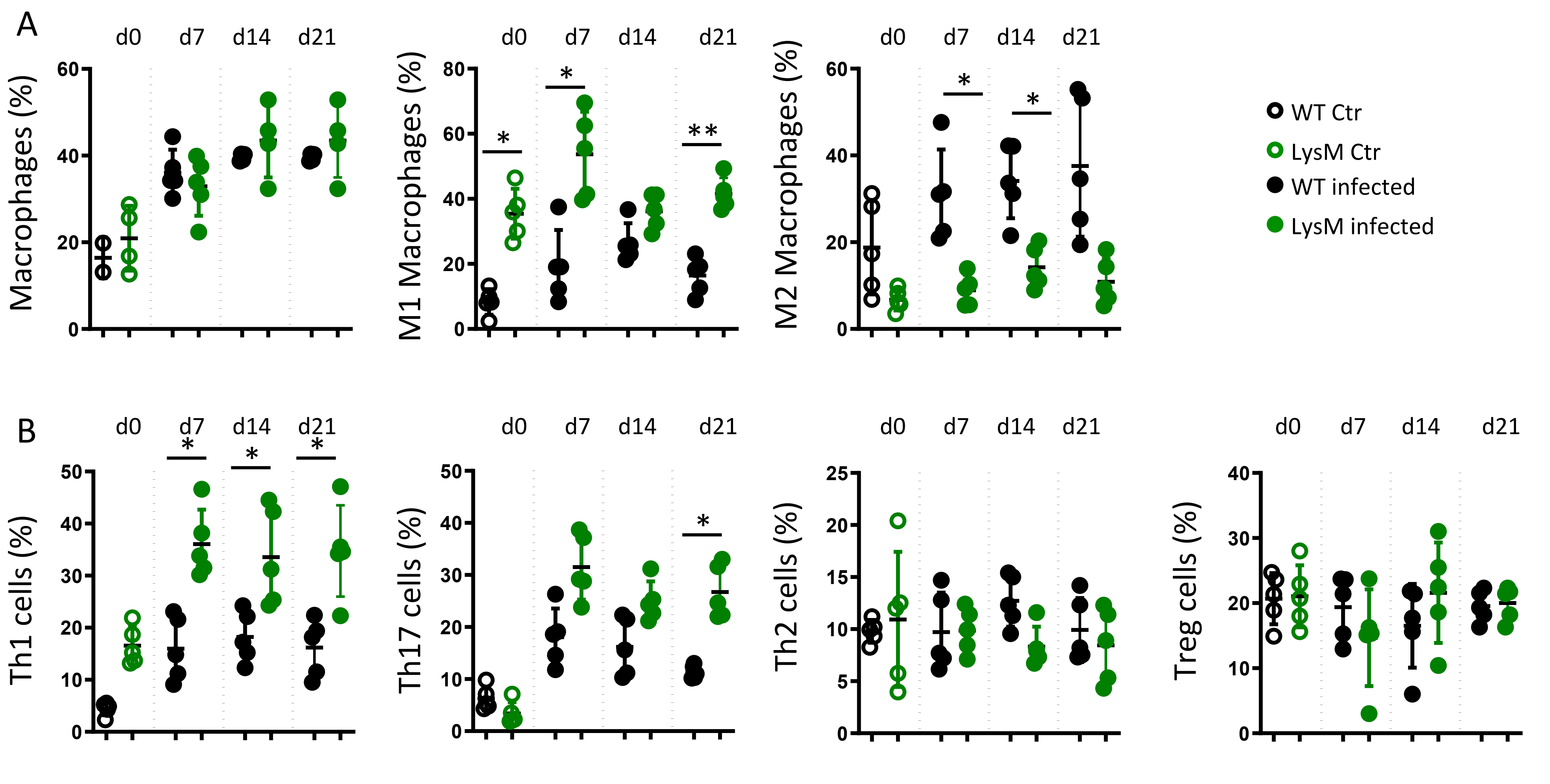

Supplementary Figure S2. Increased type 1 immune response. 8-12 week old *PTPN2*-LysMCre (LysM) mice and their *Ptpn2*$^{fl/fl}$ littermates (WT) were infected with 5x10^{8} CFU *C. rodentium.* Colon lamina propria cells were analyzed for the abundance of **A)** macrophages (life, CD45+, CD3-, B220-, CD11b+, F4/8'+, CD64+ cells), and M1 (CD86high, CD206low) and M2 (CD86low,CD206high) macrophage subsets; and **B)** abundance of Th1 (life CD45+, CD3+, IFN-g+ cells), Th17 (life CD45+, CD3+, IL-17+ cells), Th2 (life CD45+, CD3+, GATA3+ cells) or Treg (life CD45+, CD3+, FoxP3+ cells). Each dot represents an individual mouse, * = p<0.05, ** = 0.01. Related to main figures 1-2.

FIGURE S3

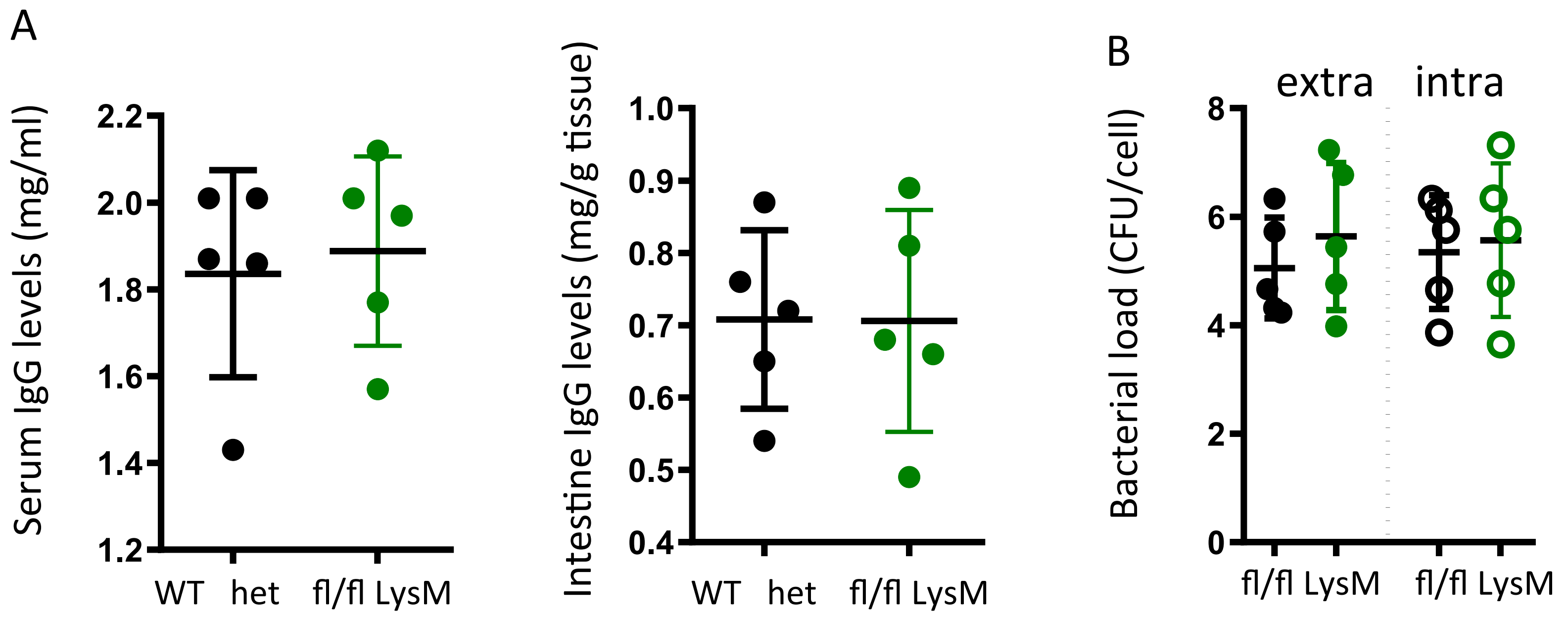

Supplementary figure S3. A) 8-12 week old *PTPN2*-LysMCre (LysM) mice and their *Ptpn2*$^{fl/fl}$ littermates (fl) were infected with 5x10^{8} CFU *C. rodentium.* IgG levels in the serum (left) and in colonic tissue (right) were assessed by ELISA. Each dot represents measurements from one individual mouse. **B)** Neutrophils were isolated from the blood of 8-12 week old *PTPN2*-LysMCre (LysM) mice and their *Ptpn2*$^{fl/fl}$ littermates (fl/fl; left graph) and infected with *C. rodentium* at an MOI of 10. After 3 h, numbers of extracellular and intracellular bacteria was determined. Each dot represents average values of triplicate measurements of cells obtained from one individual mouse, * = p<0.05

FIGURE S4

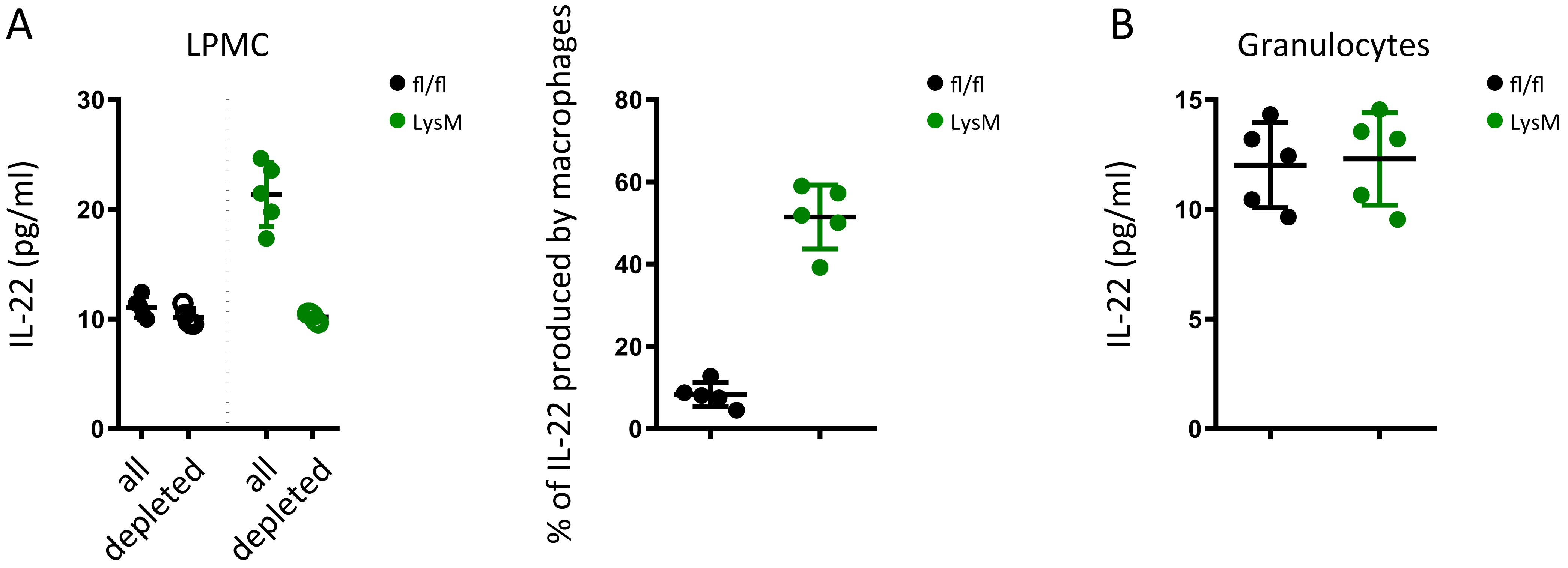

Supplementary figure S4. A) 8-12 week old *PTPN2*-LysMCre (LysM) mice and their *Ptpn2*$^{fl/fl}$ littermates (fl/fl) were infected with 5x10^{8} CFU *C. rodentium* and LPMC isolated on day 14 post infection. LPMCs (all) or macrophage-depleted LPMC (depleted) from the same mouse were cultured overnight and supernatant analyzed by ELISA for IL-22 levels. **B)** Granulocytes were isolated from WT and *Ptpn2-KO* mice and 10^{6} cells were cultured overnight prior to analysis for IL-22 in the supernatant. Related to main Figure 2.

FIGURE S5

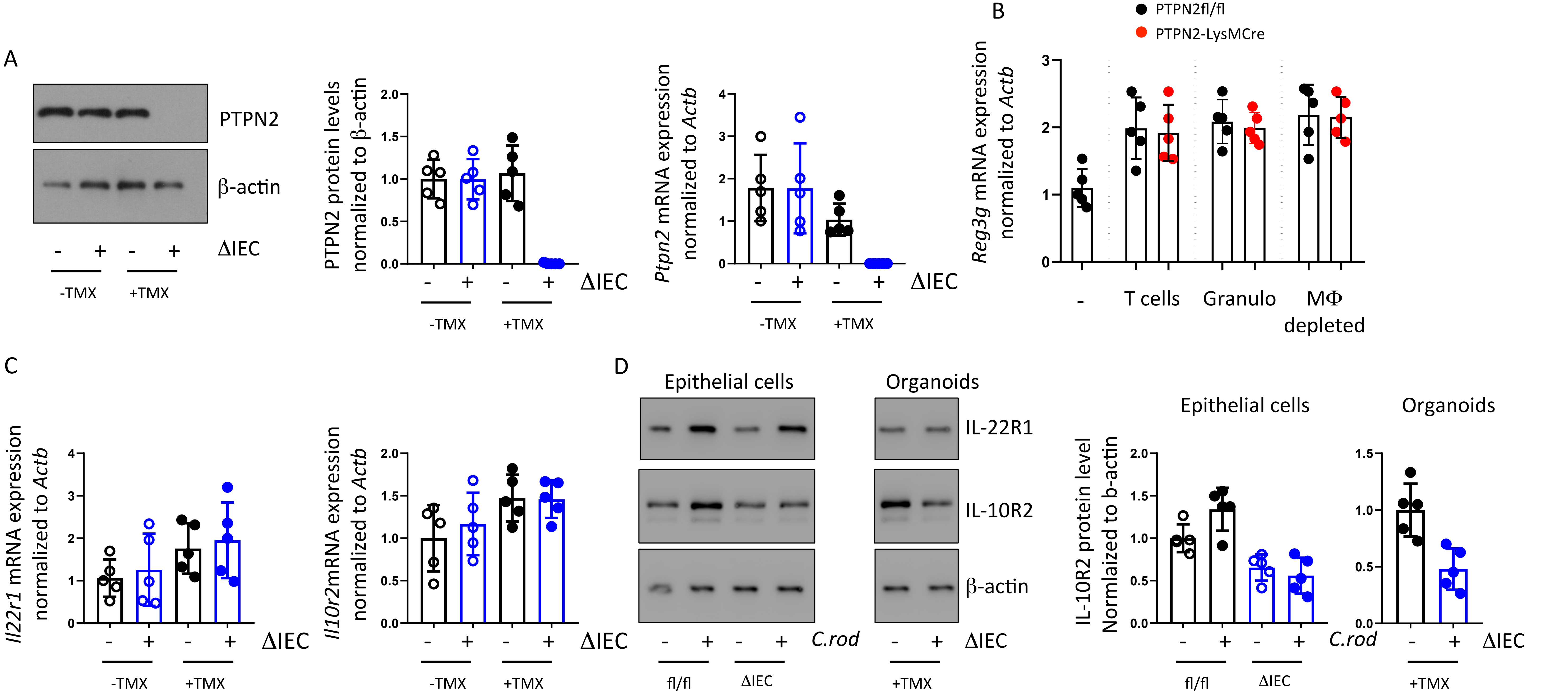

Supplementary figure S5. T cells and macrophage-depleted splenocytes from *Ptpn2*-LysMCre mice do not promote *Reg3g* mRNA expression in intestinal enteroids. A) Small intestinal organoids were derived from the small intestine of naïve *Ptpn2*^{DIEC} (VillinCre/ERT2+) or *Ptpn2*$^{fl/fl}$ littermates (VillinCre/ERT2-) and exposed to 4-hydroxy tamoxifen for 48 h to induce recombination. Tamoxifen was removed and organoids subcultured for 3 additional days prior to analysis of PTPN2 protein and mRNA expression. **B)** Small intestinal enteroids were cultured with T cells from *Ptpn2*-KO mice or macrophage depleted spleen cells from *PTPN2*-LysMCre (LysM) mice and their *Ptpn2*$^{fl/fl}$ littermates for 24 h and analyzed for mRNA expression of *Reg3g.* **C+D)** 5-8 week old *Ptpn2*^{DIEC} (DIEC) mice and their *Ptpn2*$^{fl/fl}$ littermates (WT) were injected with 1 mg/kg tamoxifen for 5 consecutive days. Four weeks later, the mice were infected with 5x10^{8} CFU *C. rodentium* and 14 days post-infection colon tissues analyzed for *Il22r1* mRNA and IL-22 protein (C) and *IL-10r2* mRNA and IL-10R2 protein (D) levels. Related to main figure 3.

FIGURE S6

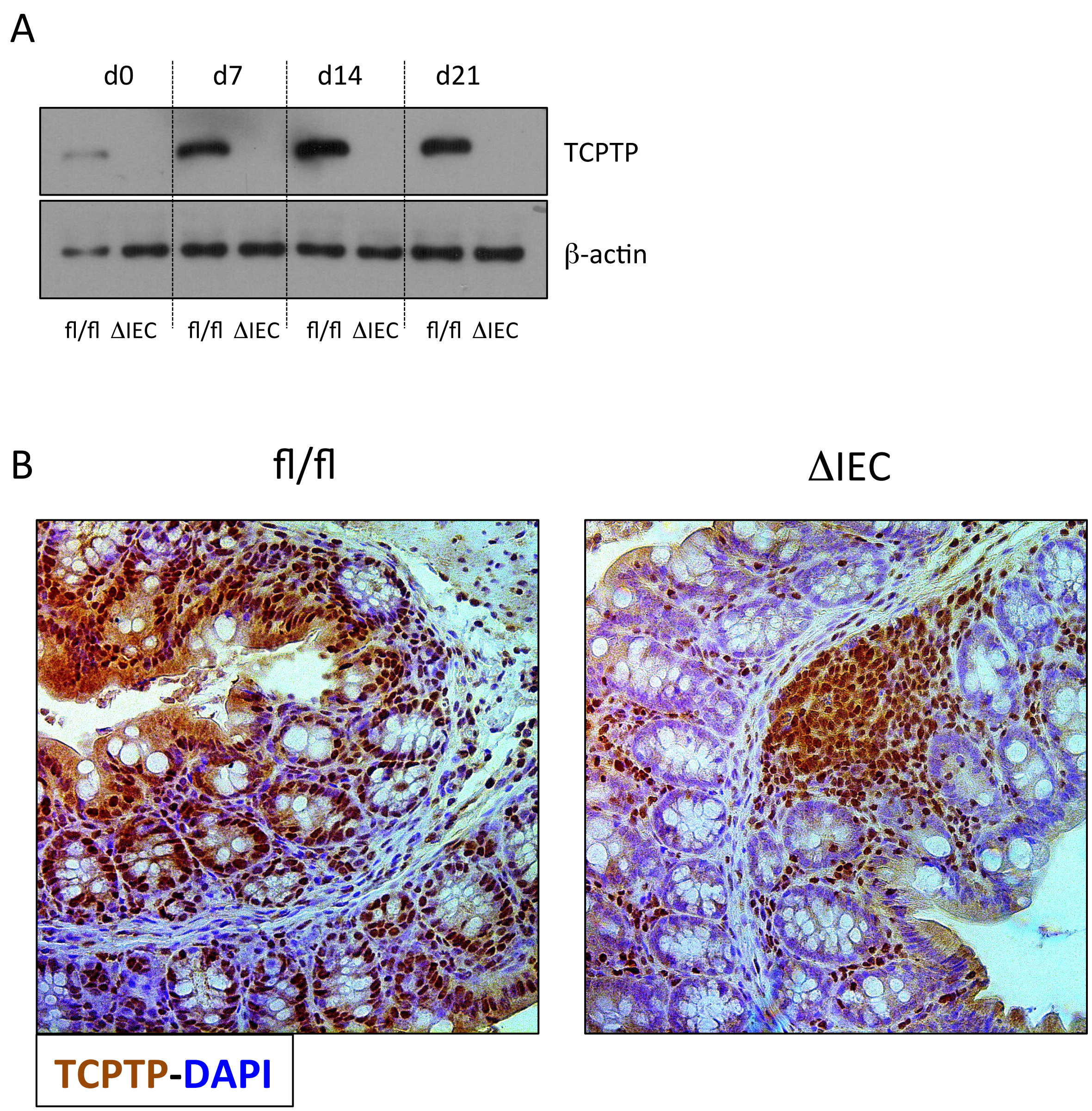

Supplementary figure S6. Verification of PTPN2 depletion in tamoxifen-treated *Ptpn2*-VilCre mice. 5-8 week old *Ptpn2*^{DIEC} (DIEC) mice and their *Ptpn2*$^{fl/fl}$ littermates (WT) were injected with 1 mg/kg tamoxifen for 5 consecutive days. Four weeks later, the mice were infected with 5x10^{8} CFU *C. rodentium* and **A)** colonic IECs, or **B)** paraffin-fixed colon pieces analyzed for PTPN2 protein expression by Western blot **(A)** or immunhistochemistry **(B)**. Related to main figure 4.

FIGURE S7

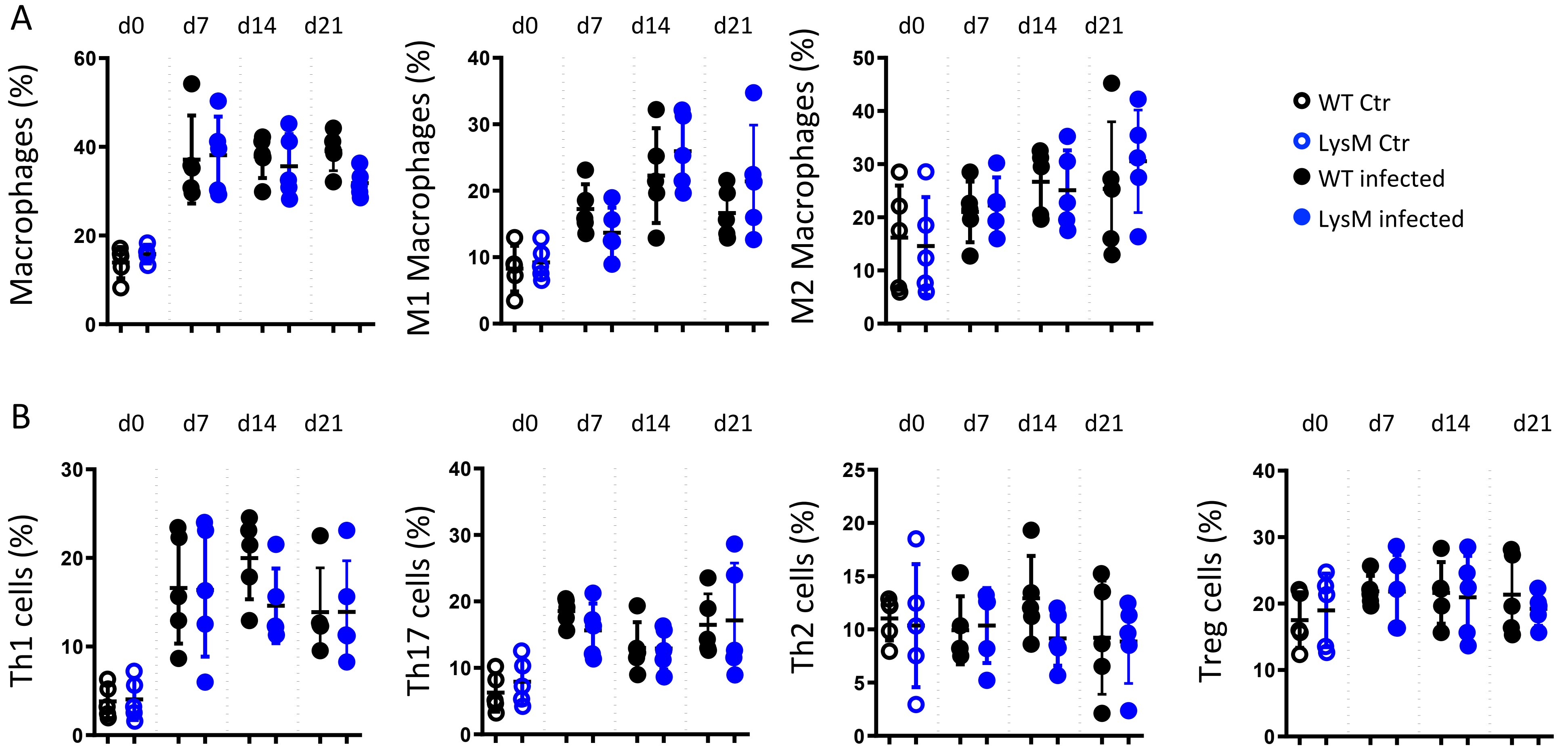

Supplementary Figure S7. No overt effect on Type 1 immune response in mice lacking PTPN2 in IECs. 5-8 week old *Ptpn2*$^{DIEC/ERT}$ (DIEC) mice and their *Ptpn2*$^{fl/fl}$ littermates (WT) were injected with 1 mg/kg tamoxifen for 5 consecutive days. Four weeks later, the mice were infected with $5x10^8$ CFU *C. rodentium.* Colon lamina propria cells were analyzed for the abundance of **A)** macrophages (life, CD45+, CD3-, B220-, CD11b+, F4/8'+, CD64+ cells), and M1 (CD86high, CD206low) and M2 (CD86low,CD206high) macrophage subsets; and **B)** abundance of Th1 (life CD45+, CD3+, IFN-g+ cells), Th17 (life CD45+, CD3+, IL-17+ cells), Th2 (life CD45+, CD3+, GATA3+ cells) or Treg (life CD45+, CD3+, FoxP3+ cells). Each dot represents an individual mouse, * = p<0.05, ** = 0.01. Related to main figure 4.

SUPPLEMENTARY FIGURE S8

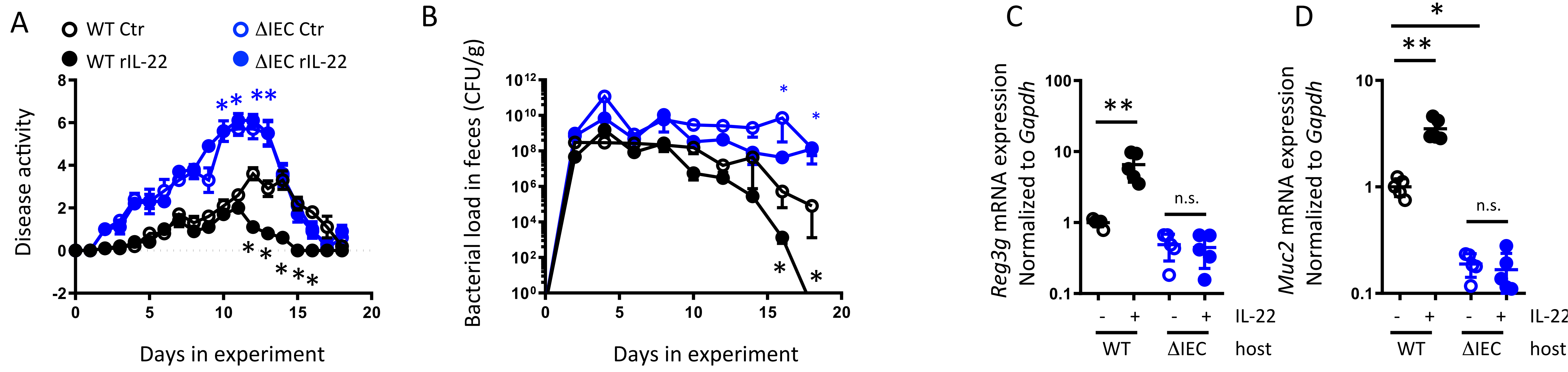

Supplementary figure S8. Supplementation of recombinant IL-22 does not ameliorate disease in *Ptpn2*-VilCre mice. 8-12 week old *Ptpn2*^{DIEC} (DIEC) mice and their *Ptpn2*$^{fl/fl}$ littermates (WT) were infected with 5x10^{8} CFU *C. rodentium.* Recombinant IL-22 (or vehicle control) was administered twice daily starting on day 3 after infection. The graphs show **A)** weight development; **B)** disease activity; and mRNA expression of **C)** *Reg3g* and **D)** *Muc2* at the end of the experiment. Each dot represents one individual mouse, * = p<0.05, ** = p<0.01. Related to main figure 4.

SUPPLEMENTARY FIGURE S9

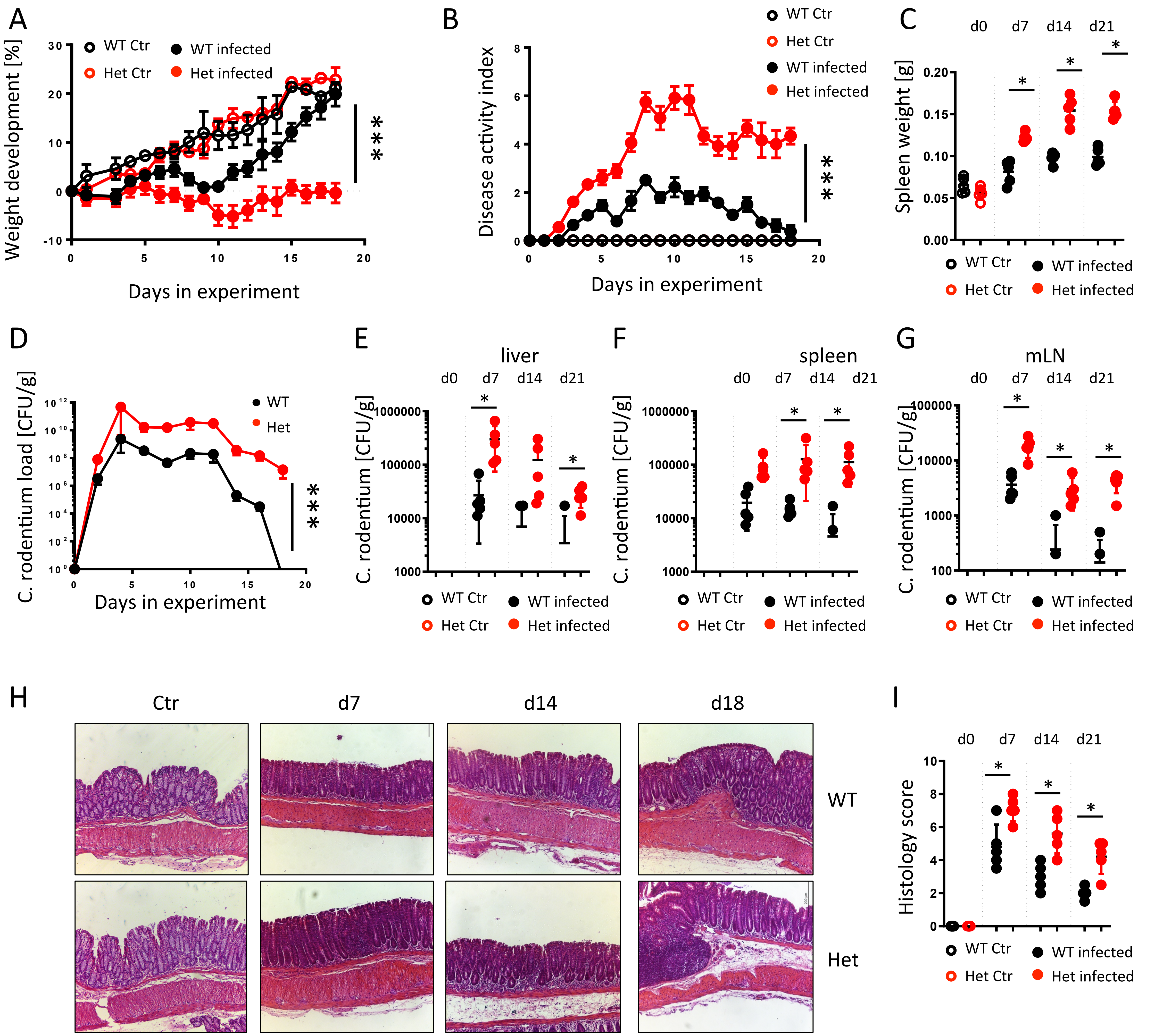

Supplementary figure S9. Whole body *Ptpn2*-Het mice are more susceptible to *C. rodentium* infection. 8-12 week old WT and *Ptpn2*-Het (Het) mice were infected with $5x10^8$ CFU *C. rodentium.* The graphs show **A)** weight development; **B)** disease activity, **C)** spleen weight on indicated days; *C. rodentium* load in **D)** feces over time, **E)** liver, **F)** spleen, and **G)** mesenteric lymph nodes (mLN) at the indicated days; **H)** representative histological pictures of the distal colon at the indicated day post infection; and **I)** scoring of damage and infiltration. Pooled data from two independent experiments with n = 5/group in total; * = p<0.05, *** = p<0.001.

FIGURE S10

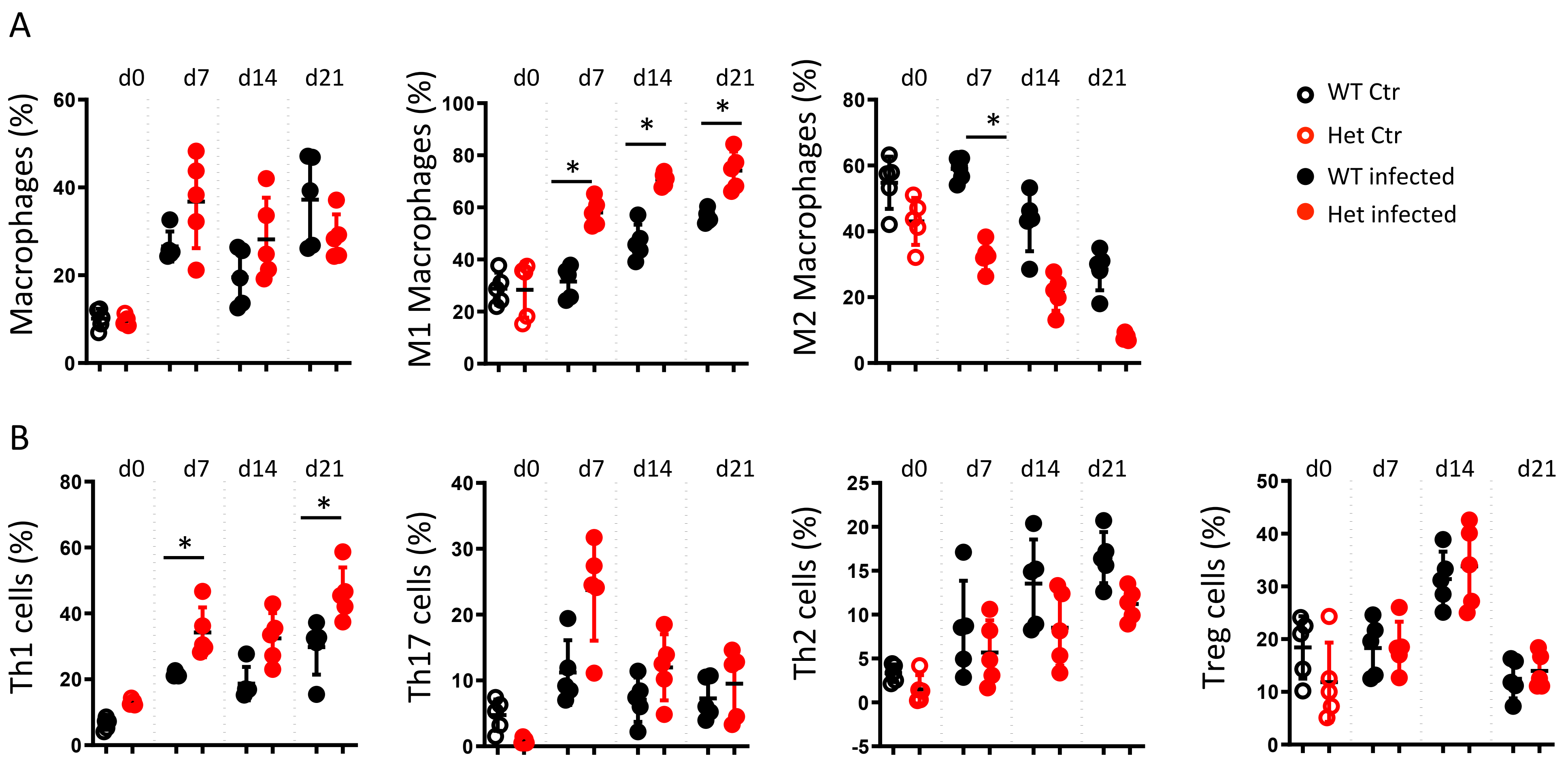

Supplementary Figure S10. Increased type 1 immune response in *Ptpn2*-Het mice. 8-12 week old *PTPN2*-Het mice and their WT littermates were infected with 5x10^{8} CFU *C. rodentium.* Colon lamina propria cells were analyzed for the abundance of **A)** macrophages (life, CD45+, CD3-, B220-, CD11b+, F4/8'+, CD64+ cells), and M1 (CD86high, CD206low) and M2 (CD86low,CD206high) macrophage subsets; and **B)** abundance of Th1 (life CD45+, CD3+, IFN-g+ cells), Th17 (life CD45+, CD3+, IL-17+ cells), Th2 (life CD45+, CD3+, GATA3+ cells) or Treg (life CD45+, CD3+, FoxP3+ cells). Each dot represents an individual mouse, * = p<0.05, ** = 0.01.

SUPPLEMENTARY FIGURE 11

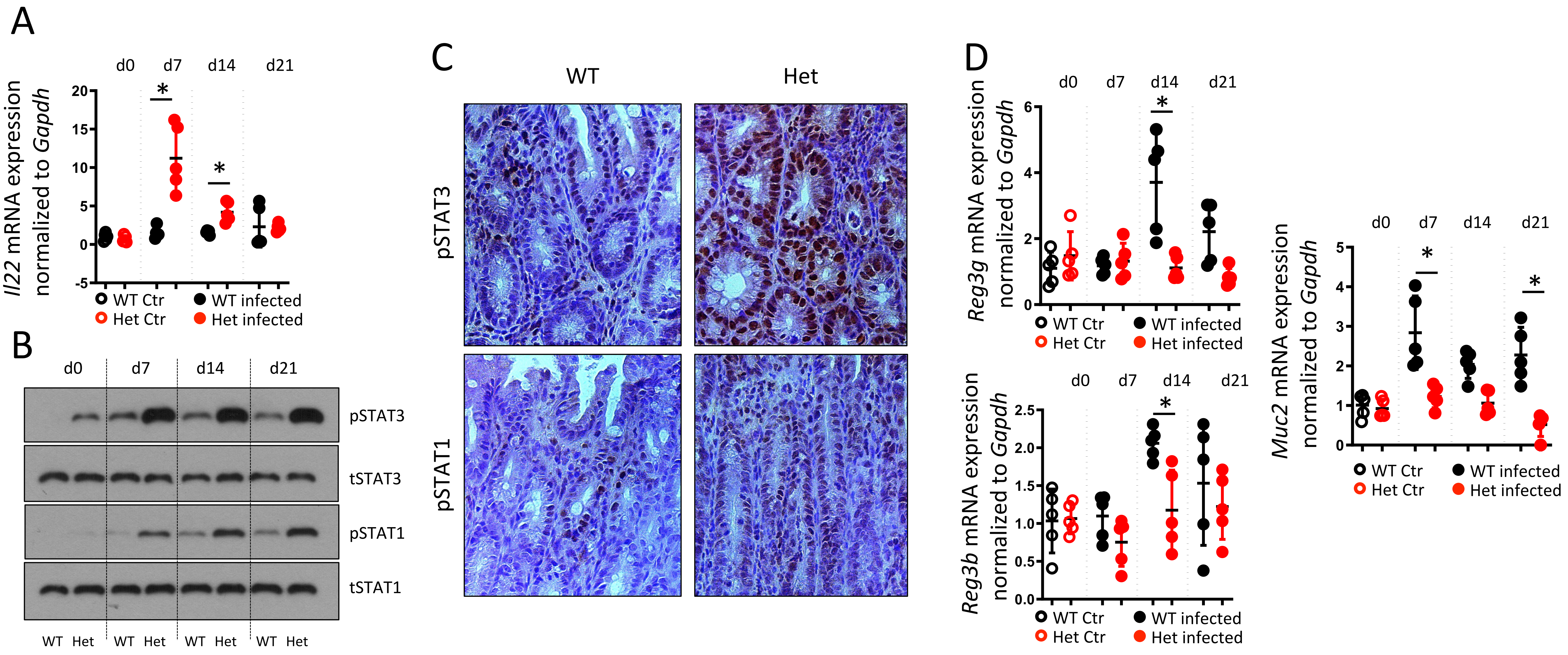

Supplementary Figure S11. Loss of *Ptpn2* in macrophages promotes IL-22 expression in the inflamed intestine and antimicrobial peptide expression in IECs. 8-12 week old WT and *Ptpn2*-Het (Het) mice were infected with $5x10^8$ CFU *C. rodentium.* **A)** *Il22* mRNA expression in the colon; **B)** phospho- and total STAT3 and STAT1 in the colon on day 7 post infection; **C)** representative images of phospho-STAT3 and phospho-STAT1 in the colon; **D:** mRNA expression of *Reg3g, Reg3b* and *Muc2* in the colon.
